# CHALLENGES OF E-INTERVENTION RESEARCH: LESSONS FROM THE ISUPPORT FOR DEMENTIA-PT FEASIBILITY STUDIES

**DOI:** 10.1093/geroni/igad104.2401

**Published:** 2023-12-21

**Authors:** Soraia Teles, Constança Paúl

**Affiliations:** University of Porto, Institute of Biomedical Sciences Abel Salazar, Porto, Porto, Portugal; University of Porto, Institute of Biomedical Sciences Abel Salazar, Porto, Porto, Portugal

## Abstract

E-interventions have become popular in delivering healthcare services, offering benefits such as increased accessibility and scalability. However, they also present ethical, technological, and research-related challenges. This poster aims to identify challenges of e-intervention research by analyzing feasibility studies of “iSupport for Dementia-PT”, an online training and support program for informal dementia caregivers (IDC) culturally adapted to Portugal from the original version by the Word Health Organization. A mixed-methods study collected data on user satisfaction and requirements for the program’s contents and interface through focus groups and usability test sessions with IDC (N=17) and health/social support professionals (N=13). A subsequent pilot study followed a mixed-methods experimental parallel between-group design with two arms (iSupport, N=21 and e-book, N=21). Ethical challenges unearthed by both studies included data privacy, e-consent reliability, equity in accessing e-health interventions, and e-health literacy. Challenging methodological decisions for an RCT protocol included defining criteria for a per protocol analysis in the context of a self-help e-intervention, timing for post-test or follow-up assessments, managing attrition, and the implications of resorting to online outcome measurements. The feasibility studies suggest that iSupport for Dementia-PT is a promising resource to support informal dementia caregivers. However, innovation is required in e-intervention research as replicating conventional procedures and methods have shown to be inadequate. Guidelines are needed on how to address the ethical, technological, and research-related challenges in e-intervention research.

